# Transauricular Vagus Nerve Stimulation in Acute Ischaemic Stroke Requiring Mechanical Thrombectomy: Sham-Controlled, Randomised Device Trial

**DOI:** 10.1007/s12975-025-01404-7

**Published:** 2025-12-27

**Authors:** Gareth L. Ackland, David Crane, Sanjali Ahuja, Amour Patel, Tim Martin, Mareena Joseph, Onika Ottley, Rizwan Hameed, Priyanthi Dias, Salma Begum, Johannes Schroth, Russ Hewson, Ana Gutierrez del Arroyo, Tom E. F. Abbott, Pervinder Bhogal

**Affiliations:** 1 Translational Medicine and Therapeutics, William Harvey Research Institute, Barts and The London School of Medicine and Dentistry, Queen Mary University of London, London, EC1M 6BQ UK; 2 Department of Interventional Neuroradiology, Royal London Hospital, Barts Health NHS Trust, London, UK

**Keywords:** Neuromodulation, Inflammation, Complications, Blood pressure, Heart rate, Vagus nerve

## Abstract

Autonomic dysfunction leads to hemodynamic instability and immunosuppression after ischaemic stroke, and is independently associated with worse outcomes. We hypothesized that non-invasive peripheral neuromodulation, using transcutaneous auricular vagal nerve stimulation (tVNS), may reduce blood pressure variability and/or reverse immunosuppression early after ischaemic stroke requiring mechanical thrombectomy (MT). In this pre-registered phase 2 study (NCT05417009), we randomized 36 patients >18 years referred for emergent MT following an acute ischaemic stroke. Patients were randomized to receive bilateral auricular active-tVNS or sham-tVNS, for the entire MT and the morning after admission. Participants, clinicians and investigators were masked to treatment allocations. The primary outcome was systolic blood pressure variability (coefficient of variation) over the entire first 24h after mechanical thrombectomy, analysed by intention-to-treat. Secondary outcomes were whole blood RNA sequencing. Explanatory measures were time/frequency-domain measures of heart rate variability. Active-tVNS was safe in this hyperacute stroke setting, with no serious adverse events recorded. The systolic blood pressure coefficient of variability over the first 24h was 0.106±0.029 after active-tVNS, compared to 0.107±0.027 after sham-tVNS (p=0.93). Active-tVNS increased the relative expression of genes coordinating tumor-necrosis factor and toll-like receptor signaling, in parallel with alterations in heart rate variability over the first 24h after MT. This phase 2 study established thatearly tVNS is safe and feasible in the hyperacute phase of acute ischaemic stroke requiring MT, but did not alter systolic blood pressure variability.

Trial registration number: NCT05417009, registered 8^TH^ June 2022.

**Supplementary Information:**

The online version contains supplementary material available at 10.1007/s12975-025-01404-7.

## Introduction

Autonomic dysfunction after acute ischaemic stroke, despite gold-standard mechanical thrombectomy therapy [1, 2], is independently associated with excess cardiovascular morbidity and all-cause mortality [3, 4]. Acute ischaemic stroke results in autonomic dysfunction characterised by a decline in parasympathetic tone, and progressive shift toward sympathetic dominance, which underpin labile systolic blood pressure variability [5]. Sympathetic hyperactivity promotes functional innate immune impairment, and high rates of infections [6]. In human neutrophils after ischemic stroke, pathogen capture through NETosis and oxidative burst in neutrophils are impaired [7].

Restoring autonomic control may limit immunosuppression and reduce post-stroke infections [8], which affect ~ 30% of patients, delay recovery and accelerate death [9]. Circulating catecholamine levels are elevated early after stroke [10], leading to β-adrenergic receptor mediated downregulation of NF-ƙB, which, in part, prevents the gene transcription of pro-inflammatory cytokines [11]. However, catecholamines do not solely explain post-stroke immunosuppression, suggesting reversing parasympathetic dysregulation through vagus nerve stimulation could also be beneficial [12].

Non-invasive vagus nerve stimulation offers a potential approach to reverse autonomic dysfunction after neurologic injury [13–15]. Stimulation of sensory fibers of the auricular branch of the vagus nerve activates the locus coeruleus-norepinephrine (LC-NE) system, mediated by the nucleus of the solitary tract [16]. Viscerosensory signals from vagal afferents are processed in a spatial and temporal manner [17], which alters efferent vagal motor activity depending on the intensity and frequency of afferent activation [18]. Autonomic neuromodulation via tVNS had been shown to confer organ protection [19] and may also reduce inflammation [20].

The potential impact of non-invasive vagus nerve stimulation on autonomic control and signatures of peripheral immunosuppression during the early phase after ischaemic stroke has not been explored in humans. In this study, we tested the hypothesis that tVNS may improve autonomic control and/or reverse immunosuppression early after ischaemic stroke requiring mechanical thrombectomy.

## Methods

### Study Design

We performed a single-center, prospective, randomized, sham-controlled phase 2 clinical trial with a double-blind intervention and a double-blind primary outcome evaluation at Royal London Hospital, Barts Health NHS Trust (London, UK), in accord with the CONSORT (Consolidated Standards of Reporting Trials) guidelines. Deidentified participant data are available from the corresponding author upon reasonable request. The trial protocol [21] and statistical analysis plan have been publicly available (https://www.qmul.ac.uk/ccpmg/media/ critical-care-and-pmg/documents/VANS-SAP-1.0). The study was approved by the research ethics committee of Health and Care Research Wales (23/WA/0013) and the UK Medicines and Healthcare Regulatory Agency (CI/2023/0006/GB). An independent Trial Monitoring Committee and steering committee oversaw trial conduct (Supplementary data).

### Participants

 We prospectively recruited patients with acute ischemic stroke referred to the Royal London Hospital service for emergent mechanical thrombectomy. We sought assent from relatives and/or written informed consent from patients after they recovered. Inclusion criteria were age>18 years, mechanical thrombectomy for acute ischaemic stroke and established hypertension and/or hypertensive on admission (defined as systolic blood pressure (BP) >140mmHg; diastolic BP >90mmHg). Exclusion criteria were participation in a trial exploring similar biological mechanism, previous enrolment into the study, anatomical contraindication (local ear abnormalities) and/or pregnancy.

### Randomization and Masking

 Patients were randomly assigned in blocks of four to receive either bilateral sham or active auricular nerve stimulation, using a secured online allocation system by the investigator performing the treatment. Randomization was not minimised. All laboratory staff were masked to clinical and allocation details.

### Intervention

**Fig. 1:**
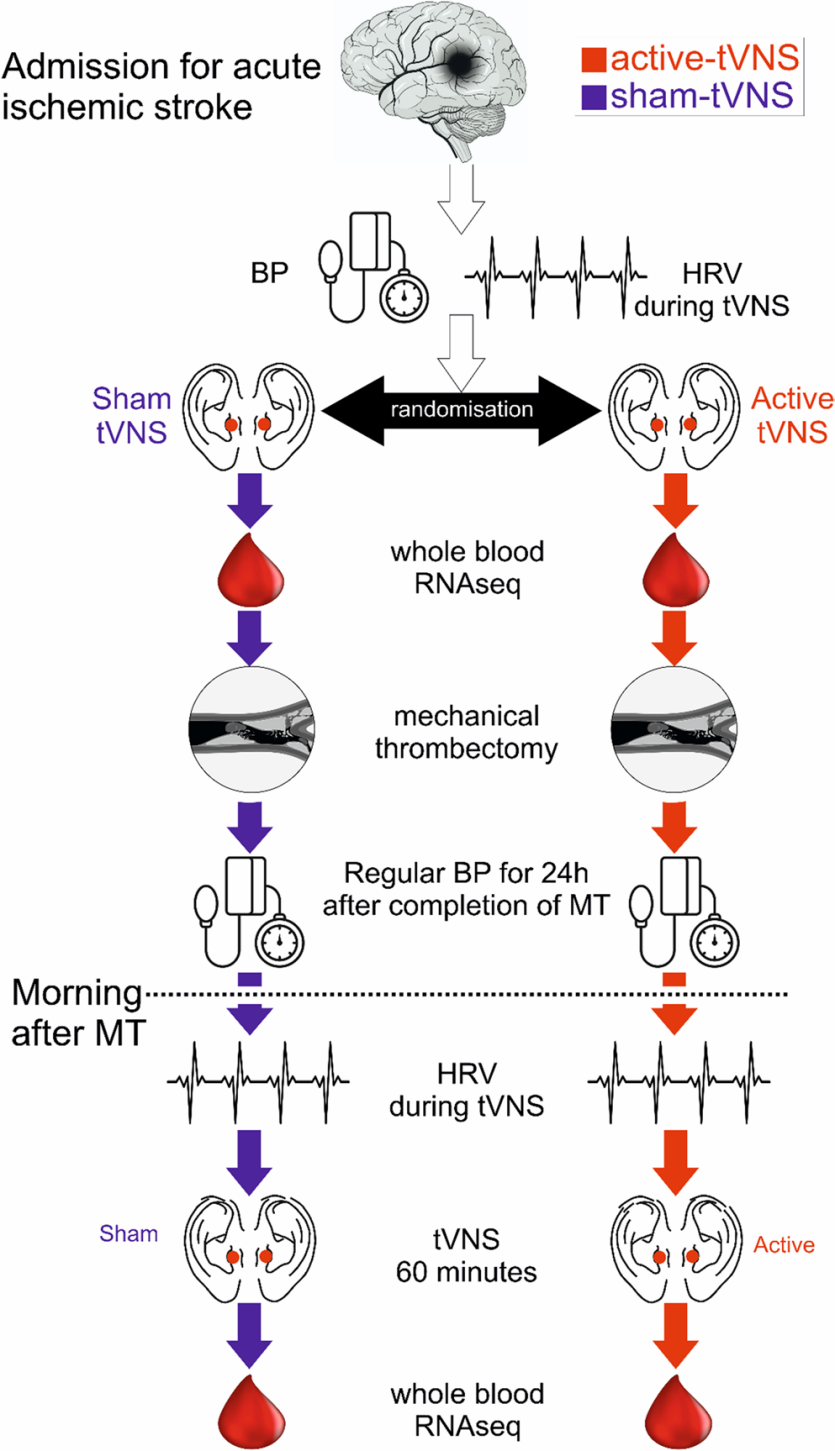
Study design

Cardiac Holter monitors (Lifecard CF, Spacelabs UK, Hertford, UK) were attached immediately to participants on arrival to the interventional radiology suite to quantify autonomic modulation of heart rate in patients in sinus rhythm (Supplementary data). Participants were randomly allocated just before arrival to the interventional radiology suite to receive either sham-tVNS or tVNS immediately after Holter monitors were placed for the duration of the mechanical thrombectomy. A further intervention period for 60 min was undertaken the morning after MT (0800–1200 h), to establish if there may be any neuromodulatory effects free of pharmacological sedation/anaesthesia. Treatment was delivered in two sessions on consecutive days (Fig. 1). Bilateral leads from an AffeX-CT/001 investigational device (Afferent Medical Solutions, Cardiff, UK) were applied to the tragi of each ear, before sedation/anaesthesia commenced and any interventional radiological interventions. This stimulation protocol was chosen based on evidence suggesting that applying sensory stimuli bilaterally is more effective than unilateral stimulations in inducing brain plasticity and repair [22, 23]. Activation of both the left and right auricular branches is similarly likely to enhance the stimulation effect, by increasing sensory input to the brainstem [24].

The stimulation current was set to 3 mA with 200 µs pulses generated at a frequency of 25 Hz [25, 26]. Pre-specified side effects were sought daily. Thereafter, bilateral sham or active-tVNS was commenced, before the induction of anaesthesia and/or sedation. Stimulation intensity was set at 3 mA for both sides, based on our literature review [26]. For sham devices, a break in the ear leads made at the time of manufacture ensured that no current was delivered. Sham or active-tVNS was delivered for the duration of the mechanical thrombectomy and again the following morning (0800–1200 h) for 1 h. To minimise any effects of sedation/anaesthesia on autonomic control, we repeated the stimulation protocol using a standardised duration of one hour the morning following MT. Because we anticipated the period of MT may be variable, the repeat stimulation period the morning after MT presented the likely best option to detect at least ECG changes during the intervention. Blood samples were obtained as soon as arterial access was obtained by the interventional radiologist, so as not to delay definitive neuroradiological intervention.

### Blood Pressure Variability 

 Arterial blood pressure was measured by oscillometry(Mindray, Huntingdon, UK), at least every 4h. Blood pressure medications were administered as per usual clinical care and not protocolised as part of the trial. The primary outcome measure was blood pressure variability over the entire first 24 h after mechanical thrombectomy, calculated as the coefficient of variation of systolic BP, which is associated with poor functional outcome in acute ischemic stroke after endovascular thrombectomy. [27] This 24 h period included the second 2nd stimulation session. Blood pressure variability may vary depending on average BP levels, so additional, pre-defined secondary outcomes for systolic blood pressure variability were calculated every 6 h over the first 24 h after admission for mechanical thrombectomy (average real variability (ARV) [28]; coefficient of variation; standard deviation, *median* absolute deviation divided by *median *value (MADM) [29].

###  Heart Rate Variability (HRV) Analysis

 Mean (SD) heart rate values captured from clinical monitors during the pre-MT, intra-MT and post-MT periods are presented. A researcher, blinded to study arm allocation, cleaned the ECG data and used Kubios HRV Premium Version 3.5.0 (Kubios, Kuopio, Finland) to derive HRV metrics from R-R intervals using both time and frequency domain analysis from the first and last 5 minutes of each recording (HRV Task Force, 1996). For both stimulation periods (i.e. during MT and the morning after MT), the effect of tVNS on HRV was compared between the first and final five minutes of each recording of the intervention period. Time-domain metrics were computed from the raw RR-interval series, whereas frequency-domain analysis was performed on a 5-Hz cubic-spline–interpolated, evenly sampled signal. Frequency domain parameters were calculated by using the autoregressive method, which offers superior spectral analysis for the shorter data sequences [30]. Spectral power, expressed as log absolute power (ms2), was quantified as power in the very low (<0.04 Hz), low (LF (0.04–0.15.04.15 Hz) and high frequency (HF) bands (0.15–0.4.15.4 Hz) [31–33]. High frequency bands reflect the influence of the vagus nerve on heart rate, whereas the low frequency band represents a complex mix of inputs from both the sympathetic and parasympathetic nervous systems [34]. 

### Clinical Outcomes

 We recorded NIH Stroke Scale (NIHSS) before and 24 h after MT and hospital-acquired infection over the first seven days after mechanical thrombectomy. We monitored and recorded pre-specified adverse events that occurred during the treatment period for the first seven days after treatment that were specific to the placement of the device (supplementary methods).

###  Systemic Inflammation

 The inflammatory status of participants at baseline was assessed using neutrophil-lymphocyte ratio, a prognostic inflammatory measure in acute ischaemic stroke [35]. The impact of the intervention on systemic inflammation was assessed by whole blood transcriptomics over the first 24 h of treatment (Illumina RNAseq, QMUL Genome Centre, London). For RNA-seq analysis, blood was collected from the femoral artery puncture site in PAXGene Blood RNA Tubes. Blood RNA tubes were immediately frozen and stored at −80°C. RNA was isolated according to the manufacturer protocol (PAXgene Blood RNA kit, PreAnalytiX, Qiagen, Hombrechtikon, Swizerland). RNA concentration and purity was measured using NanoDrop ND100 spectrophotometer (Thermo Fisher Scientific). RNA extractions and DNase-treatments were each performed in a single batch, thus limiting potential batch effects among samples during blood RNA isolation. Differential gene expression between treatment groups was evaluated by DESeq^2^ [36]. We used CIBERSORTxto estimate changes in cell type proportions over time (Supplementary methods [37]). Differential gene expression analysis was performed using Gene Specific Analysis (GSA.0.24.044) for all comparisons with a lowest coverage filter of CPM. Pathway analysis was completed using Gene Set Enrichment Analysis (GSEA.0.24.044) with the hg38 gene set database. All additional downstream analysis was performed using R v4.0.3 (Supplementary material) and iDEP, an integrated web application for differential expression and pathway analysis of RNA-Seq data [38].

### Statistical Analysis

 Normally distributed data are reported as mean (SD), while non-normally distributed data presented as median (25–75^th^ centiles). Kolmogorov-Smirnov test was used to assess normality. We estimated the sample size from the secondary analysis conducted by the BEST study, which examined blood pressure variability and neurologic outcome after endovascular thrombectomy [1]. An adjusted sample size of 36 patients was required to have a 90% chance of detecting, as significant at the 5% level, a decrease in systolic blood pressure variability from standard deviation (SD) 15±4mmHg in the sham group to SD 10±4mmHg in the actively treated group [assuming 5% non-compliance rate in each group].

 We followed the pre-specified statistical analysis plan, which was available online before data-lock (available athttps://www.qmul.ac.uk/ccpmg/sops--saps/statistical-analysis-plans-saps/). The primary outcome was blood pressure variability over the first 24 h after mechanical thrombectomy, calculated by the coefficient of variation of systolic blood pressure. The primary analysis was performed using the intention-to-treat principle, which includes all randomized participants irrespective of follow-up. Missing data were assumed to be missing at random. We performed a sensitivity analysis of the primary outcome for patients repatriated to referring hospitals within 24 h of MT and similar analyses for the effect of treatment on the secondary outcomes. Statistical analysis was performed with STATA and NCSS 2023, other than for RNAseq data. All hypotheses were tested with a 2-sided alpha of 0.05.

## Results

### Participants and Safety

**Fig. 2:**
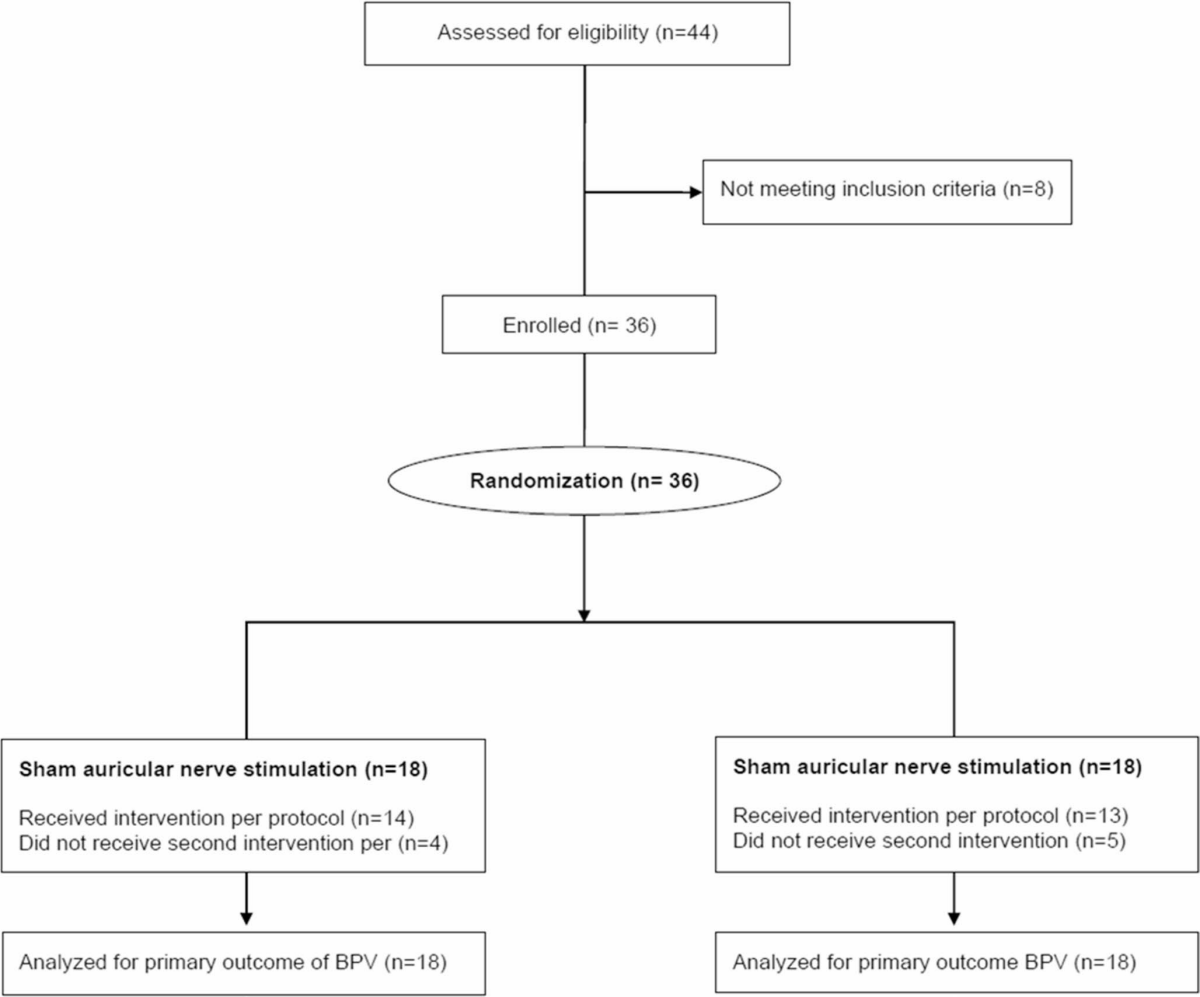
CONSORT flow chart

 Between April 26, 2023 and July 14, 2023, 44 patients confirmed to have an acute ischaemic stroke and referred for mechanical thrombectomy were screened for eligibility (Figure 2), of whom 36 were enrolled (Table 1). The severity of stroke as estimated by NIHSS on admission was similar between each group. No participants withdrew consent, but 9 patients were repatriated to their referring hospitals on the same day as the mechanical thrombectomy procedure. Four patients allocated to active-tVNS and 5 allocated to sham-tVNS did not receive the repeat intervention the morning after admission (Table 1). The mean (SD) duration from the first stimulation period to the second period was 15.4h (3.2). The median number of BP measurements over 24 h was 62 (interquartile range:39–81) in the active group, compared with 50 (37–72) in the sham group (Supplementary Figure 1).

**Table 1:** Participant characteristics and clinical management

	Sham	Active
Female sex (n; %)	9 (50%)	13 (72.2%)
Mean age, y (SD)	68.6 (15.0)	68.7 (12.7)
Last known well, h (SD)	6.6 (5.4)	7.2 (3.2)
Admission time (24 h)		
- *0000–0800 h*	3 (17%)	2 (11%)
- *0801–1800 h*	11 (61%)	13 (72%)
- *1801–2359 h*	4 (22%)	3 (17%)
Thrombus segment, MCA		
MCA, M1	14	13
MCA, M2	3	4
Other	1	1
Chronic comorbid disease - no. (%)		
COPD/asthma	3 (16.7%)	1 (5.6%)
Ischaemic heart disease	4 (22.2%)	6 (33.3%)
Diabetes mellitus	2 (11.1%)	6 (33.3%)
Heart failure	1 (5.6%)	3 (16.7%)
Active cancer	1 (5.6%)	0 (0%)
Peripheral vascular disease	1 (5.6%)	1 (5.6%)
Atrial fibrillation		
- *new atrial fibrillation*	1	0
- *established atrial fibrillation*	4	5
Current or previous smoker	5 (27.8%)	8 (44.4%)
Preoperative blood tests results		
*Hemoglobin (d/DL)*	135 (22)	142 (12)
*Creatinine (µmol/L)*	78 (44)	72 (18)
Cardiovascular medication - no. (%)		
*Beta-blocker*	9 (50.0%)	7 (38.9%)
*Calcium channel antagonist- hypertension*	4 (22.2%)	4 (22.2%)
*Doxazosin*	2 (11.1%)	1 (5.6%)
*Diuretic*	2 (11.1%)	3 (16.67%)
*Statin*	7 (38.9%)	9 (50%)
*Anti-platelet agents (aspirin*,* clopidogrel)*	5 (27.8%)	7 (38.9%)
*ACE inhibitor or ARB*	4 (22.2%)	7 (38.9%)
*SGLT2 inhibitor*	0 (0%)	1 (5.6%)
*Warfarin/DOAC*	3 (16.7%)	3 (16.7%)
*Metformin/insulin*	3 (16.7%)	3 (16.7%)
NIHSS score on admission	13 (5)	16 (7)
Troponin on admission (ng/L)	16 (12–33)	20 (14–43)
Duration of auricular nerve stimulation during thrombectomy (min)	59 (50–88)	93 (67–140)
Duration of mechanical thrombectomy (min)	51 (40–67)	80 (52–118)
Thrombectomy technique - no. (%)		
*Direct clot aspiration*	16 (88.8%)	13 (72.2%)
*Stent retrieval*	2 (11.1%)	4 (22.2%)
*Intra-arterial thrombolysis*	0 (0%)	1 (5.6%)
Anaesthetic technique - no. (%)		
*General anaesthesia*	10 (56%)	14 (78%)
*Sedation/local*	8 (44%)	4 (22%)
Cardiac arrhythmias* - no. (%)	4 (22.2%)	5 (27.8%)
Critical care on the first night after MT - no. (%)	1 (5.6%)	2 (11.1%)

Data are mean (SD) or median (25th-75th centiles); MCA M1, middle cerebral artery, sphenoidal or horizontal segment; MCA M2, middle cerebral artery, insular segment; COPD, chronic obstructive pulmonary disease; ARB – angiotensin-II receptor antagonist. *supraventricular or ventricular, as detected by attending clinicians from routine monitoring

### Clinical Outcomes

 There were two deaths within 7 days of MT in the sham-tVNS group, for whom comfort care measures were instituted within 24 h after MT. No deaths occurred after active-tVNS. Median (IQR) National Institutes of Health Stroke Scale score 24 h after active therapy was 10 (8–18), compared to 10 (5–19) for sham-tVNS. 

### Adverse Events

 One participant had local skin irritation that was not related to the site of the intervention. In addition, four participants in the sham group, and five participants in the active group, had supraventricular or ventricular cardiac arrythmias, as detected by attending clinicians from routine monitoring.

###  Primary Outcome: Blood Pressure Variability

 The coefficient of variation for systolic BP over the entire first 24 h after active-tVNS (mean (SD) was 0.106 (0.029), compared to sham-tVNS therapy 0.107 (0.027) (*p*=0.93).

### Additional Blood Pressure and Blood Pressure Variability Measurements

**Fig. 3:**
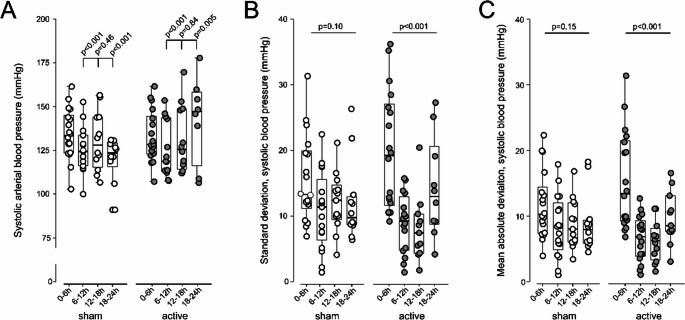
Blood pressure and blood pressure variability. **A** Systolic blood pressure over first 24 h after admission for mechanical thrombectomy, calculated as mean values over 6 h epochs (median,25–75^th^ centile box/whisker plots). P value refers to post-hoc Tukey-Kramer comparisons between measurements made during the first 6 h period and each subsequent 6 h periods. **B** Standard deviation of systolic blood pressure over first 24 h after admission for mechanical thrombectomy (median, 25–75^th^ centile box/whisker plots). **C **Median absolute deviation of systolic blood pressure over first 24 h after admission for mechanical thrombectomy (median, 25–75^th^ centile box/whisker plots). Each individual’s summary measure for the 6 h period is shown in the overlaying dot plots. P value refers to post-hoc Tukey-Kramer comparisons between first 6 h period and each subsequent 6 h period. (Supplementary table 1 for detailed data).

 Active treatment had a variable effect on systolic blood pressure during the first 24 h after mechanical thrombectomy, compared to sham-tVNS (Figure 3 A). Diastolic values were recorded but not subjected to statistical testing (Supplementary Table 1). Both additional measures of blood pressure variability, standard deviation (Figure 3B) and median absolute deviation (Figure 3 C) of systolic blood pressure were reduced by active-tVNS, compared with sham-tVNS treatments (Supplementary Tables 2, 3).

###  HeaRt Rate Variability

**Fig. 4:**
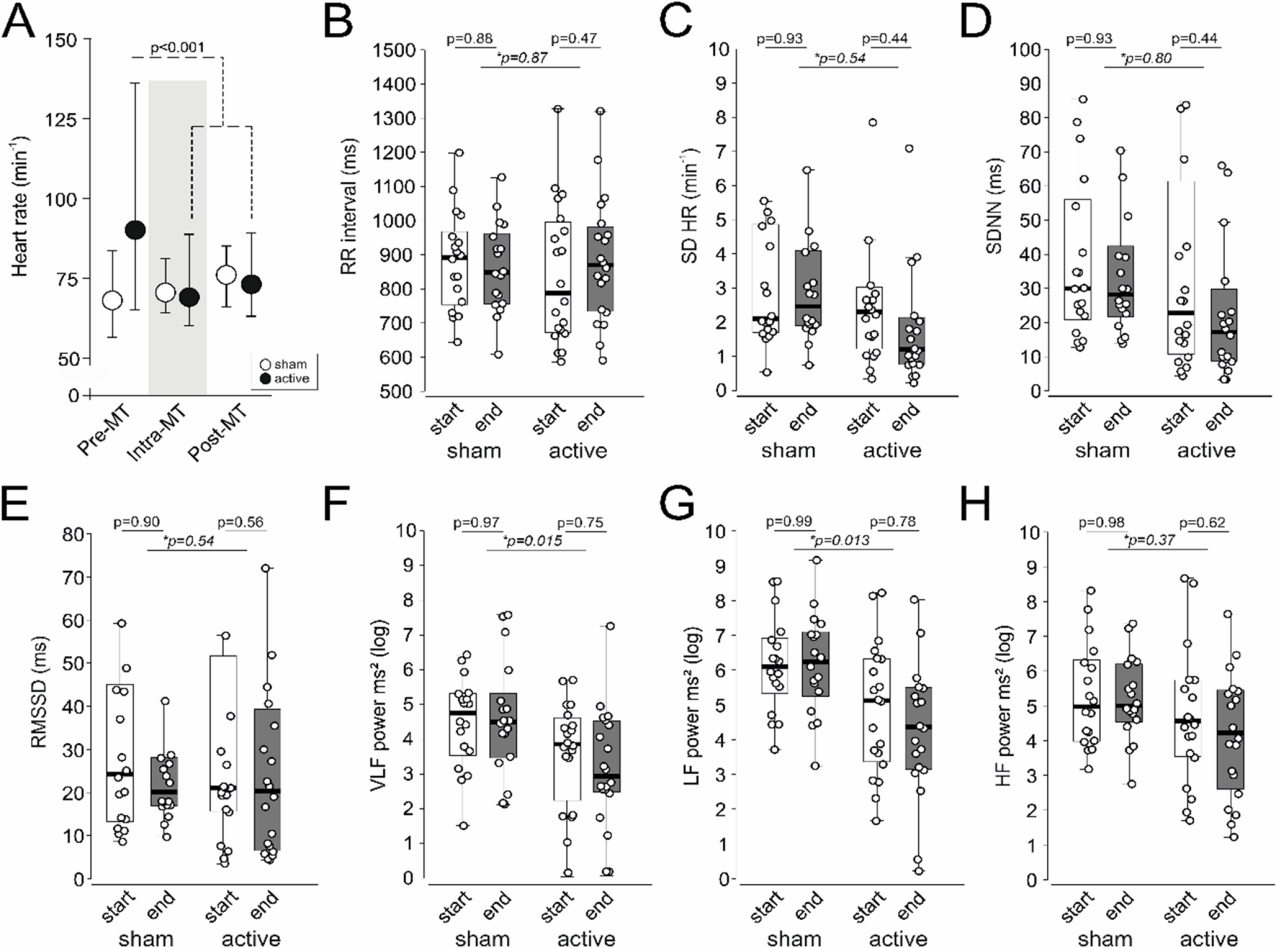
Heart rate and heart rate variability. **A** Heart rate over first 24 h after admission for mechanical thrombectomy (median,25–75^th^ centiles). P values refer to post-hoc Tukey-Kramer comparison after repeat-measures ANOVA (within group comparison) **B **RR-interval. **C** Standard deviation of heart rate (SD HR). **D** Standard Deviation of Normal-to-Normal intervals (SDNN). **E** Root Mean Square of Successive Differences (RMSSD). **F **Absolute very-low power (log scale). **G** Absolute low power (log scale). **H** Absolute high-frequency power (log scale). For panels 4B-H, box/whisker show median, 25–75^th^ centiles, with overlaying dot plots showing each individual’s summary measure for each stimulation period (both MT and morning after MT intervention periods). P values refer to within group comparison for parameter before and after each stimulation period (both MT and morning after MT intervention periods). Italicised p value with asterisk refers to comparison between sham versus active tVNS over the first 24 h of admission for MT. Additional HRV analyses are provided in Supplementary Figures 2, 3

 Ten participants with atrial fibrillation were ineligible for HRV analysis (Table 1), with complete Holter recordings for HRV analysis available from 22 participants. Heart rate was reduced by active-tVNS over the first 24 h, compared to sham-tVNS (Figure 4 A), but no differences within the stimulation period or between groups were found for time-domain measures. Over the 24 h period, active-tVNS reduced absolute power spectra for very-low frequency (mean difference in log power:1.17 (95%CI: 0.5–2.09.5.09); Figure 4 F) and low frequency (mean difference in log power:1.48 (95%CI:0.35–2.61.35.61); Figure 4G), compared to sham-tVNS.

### Systemic Inflammation

**Fig. 5:**
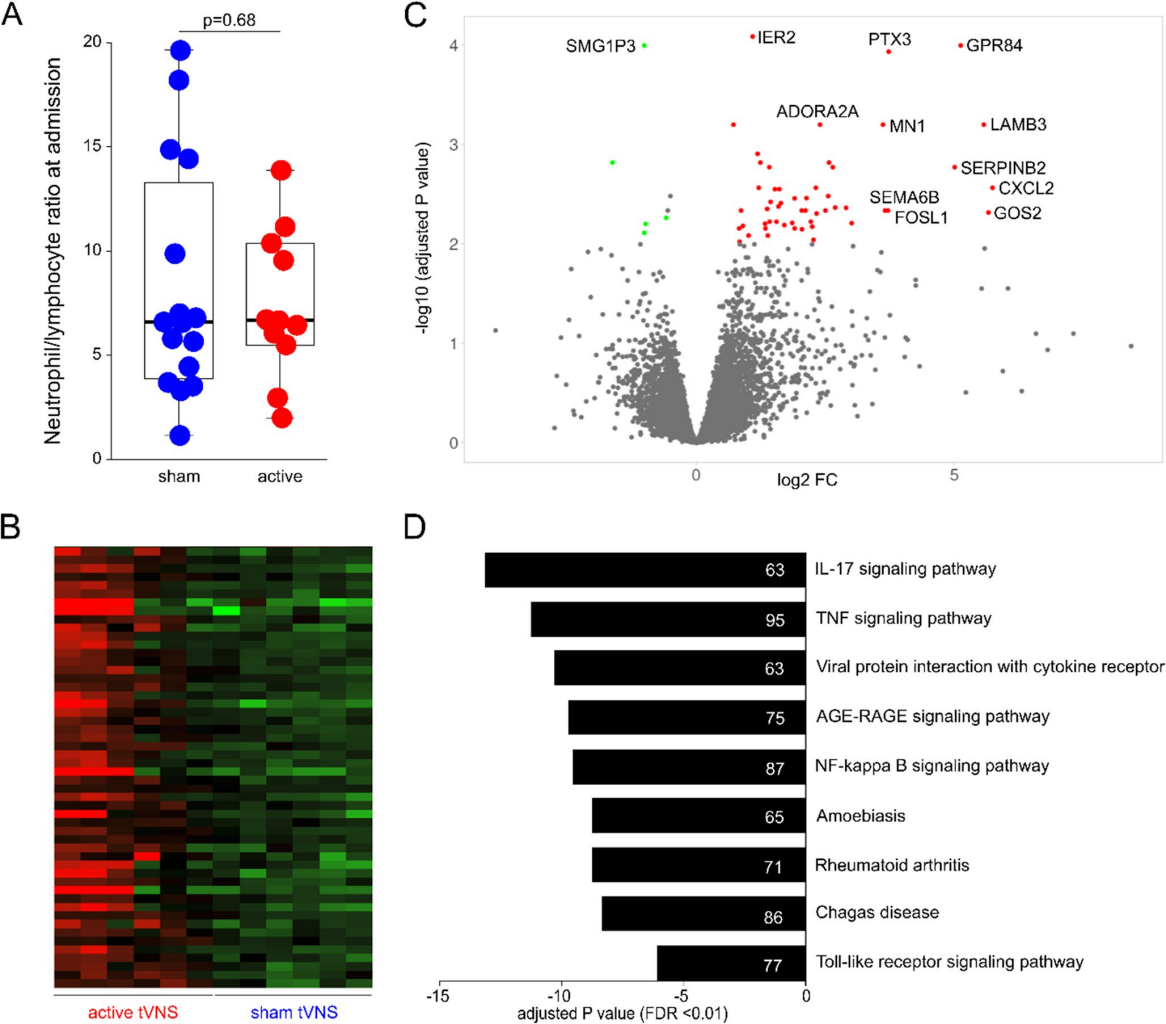
Systemic inflammation quantified by transcriptomic changes in whole blood. **A** Neutrophil/lymphocyte ratio on admission for mechanical thrombectomy. Box and whiskers plot show median, 25–75^th^ centiles. **B** Heatmap demonstrating transcriptomic changes on the morning after mechanical thrombectomy for 6 participants randomised to sham or active therapy. **C **Volcano plot depicting differentially expressed genes, of which 52 were upregulated and 5 downregulated by active tVNS (false-discovery rate <0.01; minimum fold-change ≥1.5). There were no differences in gene transcription in admission blood samples. **D** Signaling pathways significantly altered, adjusted for multiple testing with a false-discovery p-value <0.01. The number of genes differentially expressed are shown in the bars for each signaling pathway.

 The inflammatory status of participants at baseline, reflected by the neutrophil-lymphocyte ratio, was similar between the groups (mean difference: 0.8 (95% confidence interval: −3.1 to 4.8); p=0.68; Figure 5 A). From the first blood samples obtained immediately on admission, there were no differences in gene expression between participants randomised to receive active versus sham treatment (false discovery rate <0.01; minimum fold change ≥1.5). In these samples, deconvolution of the RNAseq data also confirmed similar numbers of neutrophil and lymphocyte subtypes (Supplementary figures 5–9). From samples obtained the morning after admission for mechanical thrombectomy, active-tVNS was associated with the upregulation of 52 genes and downregulation of 4 genes, correcting for a p<0.01 false-discovery rate (Figure 5B). Analysis of differentially expressed genes identified pro-inflammatory mediators to be upregulated after active stimulation, including GPR84, pentraxin-3 and adenosine A2A (Figure 5 C). KEGG pathway analysis showed that active-tVNS was associated with upregulation of TNF signalling (Figure 5D), and pathways involved in gene transcription to preserve the inflammatory response to various ligands (Supplementary figure 10).

## Discussion

 This proof of concept study confirms the safety and feasibility of undertaking transcutaneous bilateral auricular nerve stimulation in the hyperacute phase of ischaemic stroke necessitating mechanical thrombectomy. Both serial blood pressure measurements and heart rate variability recordings made during the period of stimulation were altered by tVNS, in parallel with transcriptomic signatures indicating that tVNS may have a biological effect in this setting- albeit of uncertain clinical relevance. 

 Our pilot data suggest larger trials using tVNS are feasible and safe. Restoring baroreflex control [39] and vagal activity may reduce blood pressure variability [40] which potentially limits further cardiovascular complications [41], including atrial fibrillation [42] and myocardial ischemia/infarction [43]. There is a precedent for tVNS reducing both sympathetic activity and stressor responses [44]. In nineteen volunteers who had experienced trauma (but without a diagnosis of post traumatic stress disorder), non-invasive vagus nerve stimulation decreased neural reactivity to an emotional stressor in limbic and other brain areas involved in stress [45].

 Our data from serial RNA sequencing of whole blood suggested that active tVNS increased expression of genes encoding for TNF-α signaling, which are typically supressed after middle cerebral artery occlusion in experimental murine models and humans after stroke [46]. Systemic immunosuppression after stroke is characterised by an imbalance between proinflammatory/cellular and anti-inflammatory/humoral predominant mechanisms, with a reduction of pro-inflammatory factors including TNF [47]. The proportion of monocytes capable of producing TNF-α after stroke is lower than control subjects [48]. Mechanistically, immunosuppression is at least partly caused by the prolonged over-activation of the sympathetic nervous system [49]. Given the overall between-group differences in VLF and LF components of HRV, active tVNS may attenuate sympathetic drive.

 Laboratory studies have demonstrated VNS improves neurologic function after stroke [50], in part by vagal-mediated suppression of the NLRP3 inflammasome exacerbating ischemia/reperfusion injury through neuroinflammation [51]. Both auricular and cervical vagal nerve stimulation improve behavioural performance, motor function and protect the blood brain barrier function after stroke, which is associated with lower infarct volumes [52, 53] and reduced neurologic and systemic inflammation [54]. There is substantial neurophysiologic crossover between cervical and auricular nerve stimulation [55]. Central activation of dorsal vagal motor nucleus neurons modulates peripheral inflammation without affecting the heart rate [56].

 A strength of the study was the regular recording of haemodynamic fluctuations during and after mechanical thrombectomy for up to 24h. Elevated blood pressure during the 48 hours after the onset of stroke have been associated with a lower probability of better neurologic and poor functional outcome [57, 58]. However, the unanticipated repatriation of of patients before the second intervention to their referring centre limited additional haemodynamic and inflammatory analyses, including computing ARV which is more sensitive to the number of blood pressure measurements compared with SD variability metrics [59]. The study was not designed or powered to detect favourable changes in neurologic outcome, and suffers from missing data due to early discharges, repatriation and presence of atrial fibrillation (for HRV analyses). The small numbers in this pilot study also preclude any inferences being drawn on the influence of comorbidities, duration of procedure or mode of anaesthesia. The longer duration of active-tVNS reflects the risk of small pilot studies delivering variable interventions. Additional measures of inflammation would be informative, but were not feasible for this initial project given the unpredictability in timing of when mechanical thrombectomy patients present. Our RNAseq data will now enable a targeted experimental approach using flow cytometry to further investigate the effect of active tVNS uncovered in this study. For example, the ability of peripheral immune cells to produce interleukin-6, a pleiotropic cytokine with neuroprotective properties, may be instructive given the relative restoration of TNF signalling [60]. We acknowledge that optimal tVNS settings remain unclear, particularly since experimental rodent data demonstrates that the neuronal plasticity induced by VNS is determined by an inverted-U shaped response to stimulation current but is influenced by pulse width [61].

 In summary, the early use of active-tVNS in the hyperacute phase of ischaemic stroke is feasible and may positively impact on biomarkers linked to adverse outcomes after the timely delivery of mechanical thrombectomy. The lack of harm and adverse effects attributable in this study to tVNS suggests that the further exploration of autonomic neuromodulation as a scalable, cost effective and deliverable intervention in acute ischemic stroke is warranted.

## Supplementary Information

Below is the link to the electronic supplementary material.

ESM 1 (DOCX 2.12 MB)

## Data Availability

RNA sequencing data have been deposited to the NCBI Sequence Read Archive. The data that support the findings of this study are available upon written request. This study receives funding support from the UKRI and adheres to its data sharing policies.
