# Supplementary material for: Transauricular Vagus Nerve Stimulation in Acute Ischaemic Stroke Requiring Mechanical Thrombectomy: Sham-Controlled, Randomised Device Trial
Source: Transl Stroke Res. 2025 Dec 27;17(1):10. doi: 10.1007/s12975-025-01404-7 (PMC12743696; doi:10.1007/s12975-025-01404-7)
Supplement: Supplementary file 1 — (DOCX 2.12 MB) [file 12975_2025_1404_MOESM1_ESM.docx]

**Transauricular vagus nerve stimulation in acute ischaemic stroke requiring mechanical thrombectomy: phase 2A sham-controlled randomised device trial.**

SUPPLEMENTARY INFORMATION

Contents

[Pre-defined adverse events related to device placement 2](#_Toc215502698)

[Measures of blood pressure variability 2](#_Toc215502699)

[RNA sequencing 2](#_Toc215502700)

[Analysis of RNA-sequencing data 3](#_Toc215502701)

[Bioinformatic workstream for RNAseq analysis. 4](#_Toc215502702)

[Cell-type deconvolution. 4](#_Toc215502703)

[R code for DSEQ1 analysis. 5](#_Toc215502704)

[Supplementary Table 1. Cardiovascular medication after MT. 7](#_Toc215502705)

[Supplementary Table 2. Diastolic blood pressure over first 24h after mechanical thrombectomy 7](#_Toc215502706)

[Supplementary Table 3. SD and MADM within treatment group comparisons (Tukey Kramer post hoc testing, time x treatment interaction). 8](#_Toc215502707)

[Supplementary Figure 1. Median (IQR) number of BP readings made during 24h after admission for MT. 9](#_Toc215502708)

[9](#_Toc215502709)

[Supplementary Figure 2. Proportion of LF and HF power after active versus sham treatment. 9](#_Toc215502710)

[Supplementary Figure 3. Poincare SD2 SD1 plot, after active versus sham treatment. 10](#_Toc215502711)

[Supplementary Figure 4. Principal component analysis. 11](#_Toc215502712)

[Supplementary Figure 5. Serial neutrophil-lymphocyte ratio. 12](#_Toc215502713)

[Supplementary Figure 6. Differential cell counts on admission immediately before MT. 12](#_Toc215502714)

[Supplementary Figure 7. neutrophil types determined by CIBERSORT. 13](#_Toc215502715)

[Supplementary Figure 8. lymphocyte populations determined by CIBERSORT. 14](#_Toc215502716)

[Supplementary Figure 9. monocytes, eosinophils and platelets quantified by CIBERSORT. 15](#_Toc215502717)

[Supplementary Figure 10. pathway analysis by KEGG 16](#_Toc215502718)

[TNF signaling 16](#_Toc215502719)

[IL-17 signalling pathway 17](#_Toc215502720)

[NOD-like receptor signalling pathway 18](#_Toc215502721)

[Toll-like receptor signalling pathway 18](#_Toc215502722)

[Staphylococcus aureus infection 19](#_Toc215502723)

[Cytosolic DNA-sensing pathway 20](#_Toc215502724)

[Additional references 21](#_Toc215502725)

# Pre-defined adverse events related to device placement

- - Light-headedness
  - fatigue/tiredness
  - Mood changes
  - Neck pain
  - Tooth pain
  - Pain/local skin irritation due to attachment of the device ear clips
  - Tingling sensation due to the use of the device
  - Palpitations

# Measures of blood pressure variability

The average real variability (ARV) is more dependent on the number of ambulatory BP readings than the standard deviation. We therefore calculated the coefficient of variation of systolic BP plus standard deviation, median absolute deviation divided by median value (MADM) as additional pre-defined measures of systolic blood pressure variability calculated every 6h over the first 24h after admission for mechanical thrombectomy.

# RNA sequencing

Blood samples (0 mL) were collected into citrated bottles and transferred to PAXgene™ tubes (PreAnalytiX, Switzerland) for RNA stabilization, and stored at -80°C until assayed. Total RNA was extracted using the PAXGene RNA extraction kit (Qiagen, Hilden, Germany) according to the manufacturer’s instructions, with a DNase step included to remove contaminating DNA. RNA samples were assessed for quantity and integrity using the NanoDrop 8000 spectrophotometer v2.0 (ThermoScientific, USA) and Agilent 200 Bioanalyser (Agilent Technologies, Waldbronn, Germany), respectively. All samples that displayed a RIN score of 6.5 or higher were used for RNA library preparation (NEBNext Globin & rRNA Depletion Kit for Human/Mouse/Rat). Samples were randomised before library preparation and sequencing using NextSeq2000 P3 00-cycle kit (Illumina Inc., Cambridge, UK).

# Analysis of RNA-sequencing data

RNA-sequencing data generated was first analysed using Partek Flow Software v.0. Pre-alignment quality control was completed using FastQC. rDNA, tRNA and mtrDNA reads were filtered out using Bowtie2 (v2.2.5). Data was aligned to genome build hg38 using Spliced Transcripts Alignment to a Reference (STAR) v2.7.3a. Aligned reads were quantified to the annotation model Ensembl Transcripts release 09, with the reverse-forward strand specified. Gene counts were normalised using counts per million (CPM) + 0.000. Principal Component Analysis was performed, and the first 25 principal components were calculated. Differential gene expression analysis was performed using Gene Specific Analysis (GSA .0.24.044) for all comparisons with a lowest coverage filter of CPM. Pathway analysis was completed using Gene Set Enrichment Analysis (GSEA .0.24.044) with the hg38 gene set database. Samtools v. and bcftools v. was used to detect variants with a Min gapped fraction of 0. RNA sequence data has been deposited at the European Genome-phenome Archive (EGA). All additional downstream analysis was performed using R v4.0.3 and iDEP, an integrated web application for differential expression and pathway analysis of RNA-Seq data. R packages fgsea, and MSigDB v7.4. including canonical pathways (KEGG) and Gene Ontology (GO) terms were utilized for gene-set enrichment analysis (GSEA) by hypergeometric test.

# Bioinformatic workstream for RNAseq analysis.


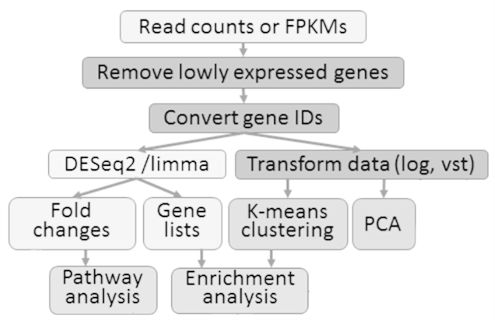


# Cell-type deconvolution.

Cell-type deconvolution was performed using CIBERSORTx. [12] Cell type specific gene expression from the Kwok et. al. single cell RNA-Seq dataset was obtained by down sampling the CPM normalised data to 00 cells per population.[13] The CIBERSORTx fractions module was then used to generate a signature matrix and estimate cell population fractions from the CPM normalised RNA-Seq data, using S-mode batch correction and the following parameters; fraction = 0.25 and replicates = 00.

# R code for DSEQ1 analysis.

# R version 4.4.0 (2024-04-24)

if (!require("BiocManager", quietly = TRUE))

install.packages("BiocManager") # v. 1.30.23

if (!require("DESeq2", quietly = TRUE))

BiocManager::install("DESeq2") # v. 1.44.0

library(DESeq2) # D.E.G.

FC <- 1.49 # Fold-change cutoff

FDR <- 0.01 # FDR cutoff

alpha <- 0.1 # independent filtering, default

# Prepare data --------------------

# Use the "Converted counts" button in the Pre-Process tab

# to download the filtered counts file with gene IDs converted to Ensembl.

raw_counts = read.csv("converted_counts_data.csv")

row.names(raw_counts) <- raw_counts$User_ID

raw_counts <- raw_counts[, -(1:3)] # delete 3 columns of IDs

str(raw_counts)

# Factors coded: treatment --> A

col_data <- data.frame(

"A" = c("SHAM", "SHAM", "SHAM", "SHAM", "SHAM", "SHAM", "ACTIVE", "ACTIVE", "ACTIVE", "ACTIVE", "ACTIVE", "ACTIVE")

)

row.names(col_data) <- colnames(raw_counts)

col_data

#Set reference level

col_data[, 1] <- as.factor(col_data[, 1])

col_data[, 1] <- relevel(col_data[, 1], "SHAM")

# Run DESeq2--------------------

dds <- DESeq2::DESeqDataSetFromMatrix(

countData = raw_counts,

colData = col_data,

design = ~ A

)

dds = DESeq2::DESeq(dds)

# Extract results--------------------

# Comparison 1 of 1: ACTIVE-SHAM

res <- DESeq2::results(dds,

contrast = c("A", "ACTIVE", "SHAM"),

independentFiltering = TRUE,

alpha = alpha

)

# Examine results

summary(res)

plotMA(res)

plotCounts(dds, gene = which.min(res$padj), intgroup = colnames(col_data)[2])

res <- subset(res, padj < FDR & abs(log2FoldChange) > log2(FC)) # Select

table(sign(res$log2FoldChange)) # N. of genes Down, Up

res <- res[order(-res$log2FoldChange), ] #sort

head(res) #top upregulated

tail(res) #top downregulated

# Supplementary Table 1. Cardiovascular medication after MT.

N, % values are shown, with number of participants remaining in hospital the morning after MT [i.e. not repatriated]

| Cardiovascular medication - no. (%) | active | sham |
| --- | --- | --- |
| *Beta-blocker* | 1/12 | 3/13 |
| *Calcium channel antagonist- hypertension* | 0/12 | 3/13 |
| *Doxazosin* | 0/12 | 1/13 |
| *Diuretic* | 0 | 0 |
| *Statin* | 2/12 | 4/13 |
| *Nitrate* | 3/12 | 2/13 |
| *ACE inhibitor or ARB* | 1/12 | 1/13 |

# Supplementary Table 2. Diastolic blood pressure over first 24h after mechanical thrombectomy

Summary values for diastolic blood pressure following tVNS. Abbreviations: MADM- median absolute deviation of diastolic blood pressure; CoV- coefficient of variability of diastolic blood pressure.

| **Mean diastolic BP** | **0-6h** | **6-12h** | **12-18h** | **18-24h** |
| --- | --- | --- | --- | --- |
| *sham* | 75 | 79 | 72 | 68 |
| *active* | 78 | 75 | 68 | 64 |
| **Standard deviation diastolic BP** |  |  |  |  |
| *sham* | 14 | 14 | 12 | 11 |
| *active* | 20 | 13 | 16 | 15 |
| **MADM diastolic BP** |  |  |  |  |
| *sham* | 11.06 | 11.54 | 9.21 | 8.99 |
| *active* | 16.27 | 10.34 | 13.45 | 11.86 |
| **CoV diastolic BP** |  |  |  |  |
| *sham* | 0.19 | 0.18 | 0.17 | 0.16 |
| *active* | 0.26 | 0.18 | 0.24 | 0.23 |

# Supplementary Table 3. SD and MADM within treatment group comparisons (Tukey Kramer post hoc testing, time x treatment interaction).

|  | **Count** | **Mean SD** | **Difference** | **P-Value** |
| --- | --- | --- | --- | --- |
| active, 0-6h | 18 | 19.5 |  |  |
| - active, 6-12h | 13 | 10.2 | 9.3 (1.8 to 16.7) | 0.005 |
| - active, 12-18h | 10 | 9.8 | 9.7 (1.7 to 17.8) | 0.008 |
| - active, 18-24h | 8 | 14.6 | 4.9 (-3.8 to 13.5) | 0.634 |
|  |  |  |  |  |
| sham, 0-6h | 18 | 16.2 |  |  |
| - sham, 6-12h | 14 | 12.5 | 3.7 (-3.6 to 10.9) | 0.730 |
| - sham, 12-18h | 12 | 11.2 | 5.0 (-2.6 to 12.6) | 0.442 |
| - sham, 18-24h | 7 | 8.6 | 7.6 (-1.5 to 16.7) | 0.165 |

|  | **Count** | **Mean MADM** | **Difference** | **P-Value** |
| --- | --- | --- | --- | --- |
| active, 0-6h | 18 | 14.8 |  |  |
| - active, 6-12h | 13 | 7.3 | 7.6 (2.5 to12.6) | <0.0001 |
| - active, 12-18h | 10 | 6.6 | 8.3 (3.1 to 13.4) | <0.0001 |
| - active, 18-24h | 8 | 9.7 | 5.1 (-0.8 to 11.0) | 0.140 |
|  |  |  |  |  |
| sham, 0-6h | 18 | 12.3 |  |  |
| - sham, 6-12h | 14 | 9.6 | 2.7 (-2.2 to 7.5) | 0.666 |
| - sham, 12-18h | 12 | 8.4 | 3.9 (-1.2 to 9.1) | 0.270 |
| - sham, 18-24h | 7 | 7.4 | 4.9 (-0.5 to 10.4) | 0.107 |

# Supplementary Figure 1. Median (IQR) number of BP readings made during 24h after admission for MT.

#
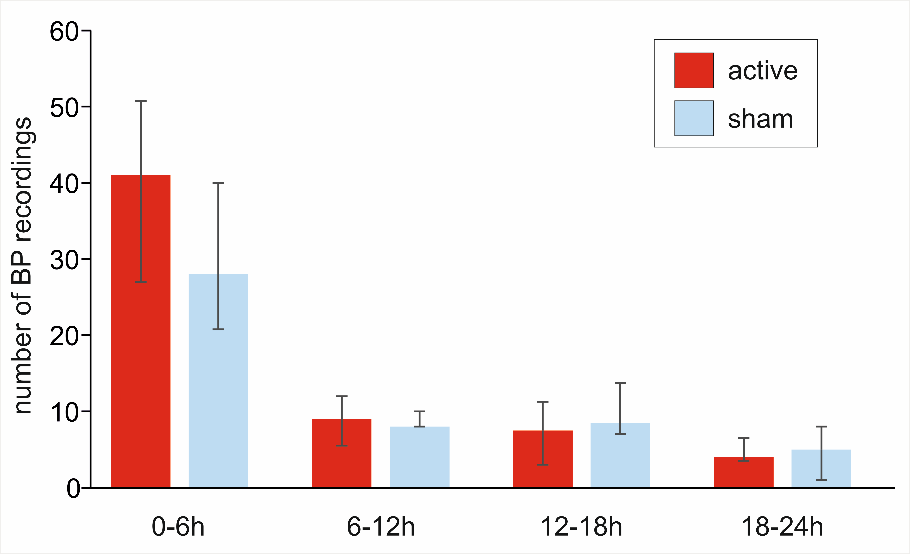


# Supplementary Figure 2. Proportion of LF and HF power after active versus sham treatment.


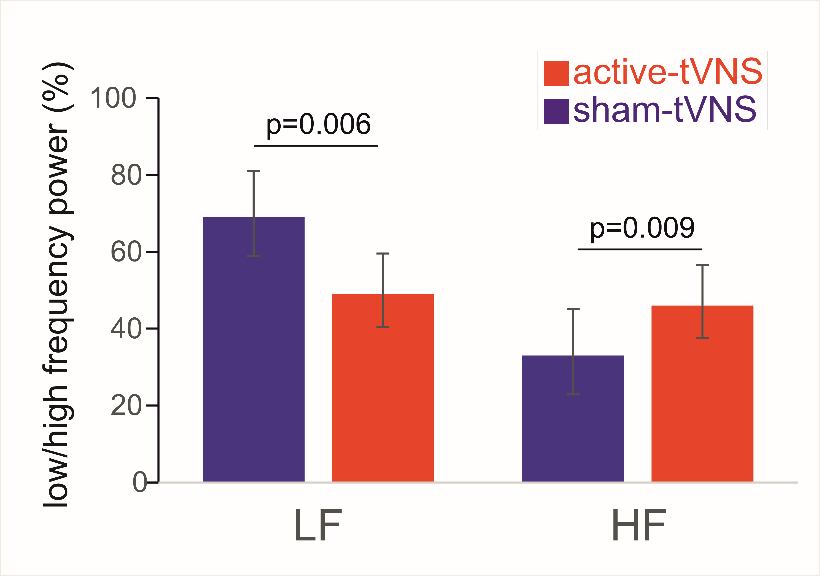
Proportion of LF/ HF power contributing to cardiac autonomic modulation over first 24h after admission for mechanical thrombectomy. P values refer to post-hoc Tukey-Kramer comparison after repeat-measures ANOVA (between active versus sham-tVNS group comparison)

# Supplementary Figure 3. Poincare SD2 SD1 plot, after active versus sham treatment.


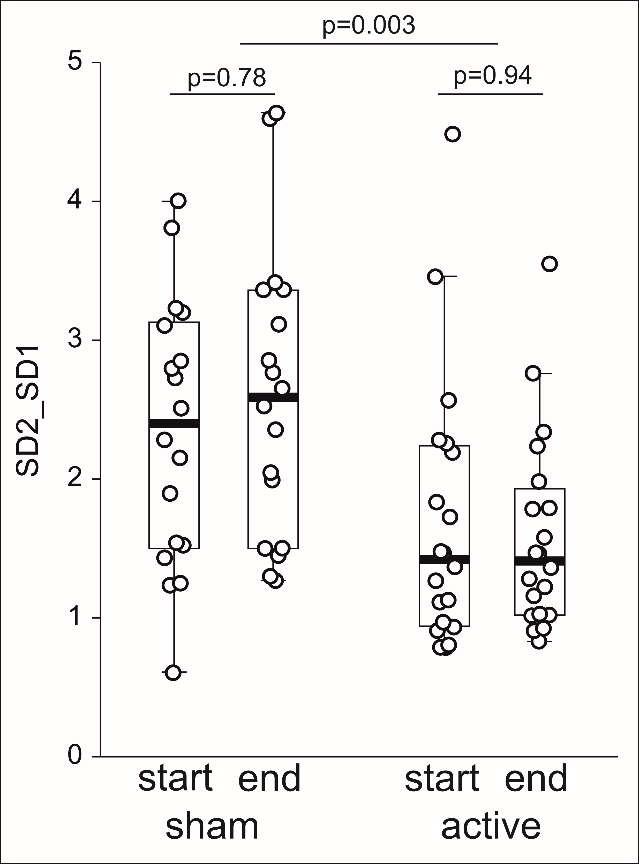
SD2/SD1 ratio reflects the sympathovagal balance, with a lower ratio reflecting more short-term variability and stronger parasympathetic (vagal) tone. No significant changes between start and end stimulation period, but a difference between sham and active at the end of the study suggests the active treatment produced a different autonomic balance compared with sham.

# Supplementary Figure 4. Principal component analysis.

All additional downstream analysis was performed using R v4.0.3 and iDEP, an integrated web application for differential expression and pathway analysis of RNA-Seq data including principal component analysis.


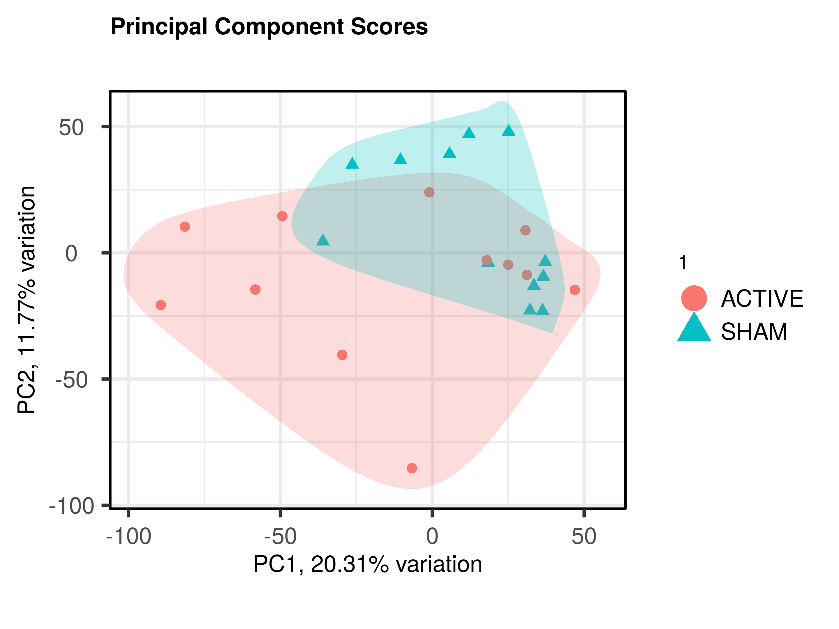


# Supplementary Figure 5. Serial neutrophil-lymphocyte ratio.


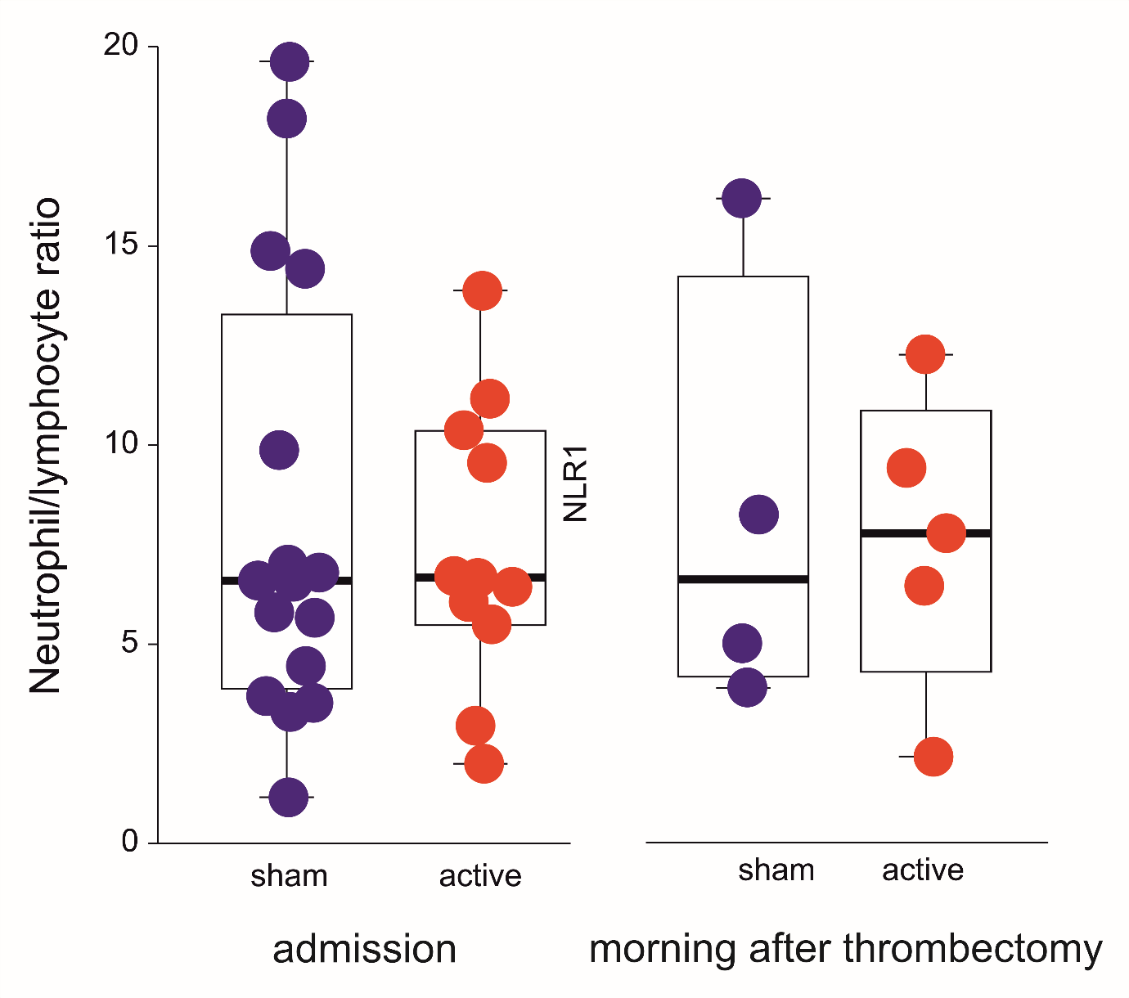


# Supplementary Figure 6. Differential cell counts on admission immediately before MT.


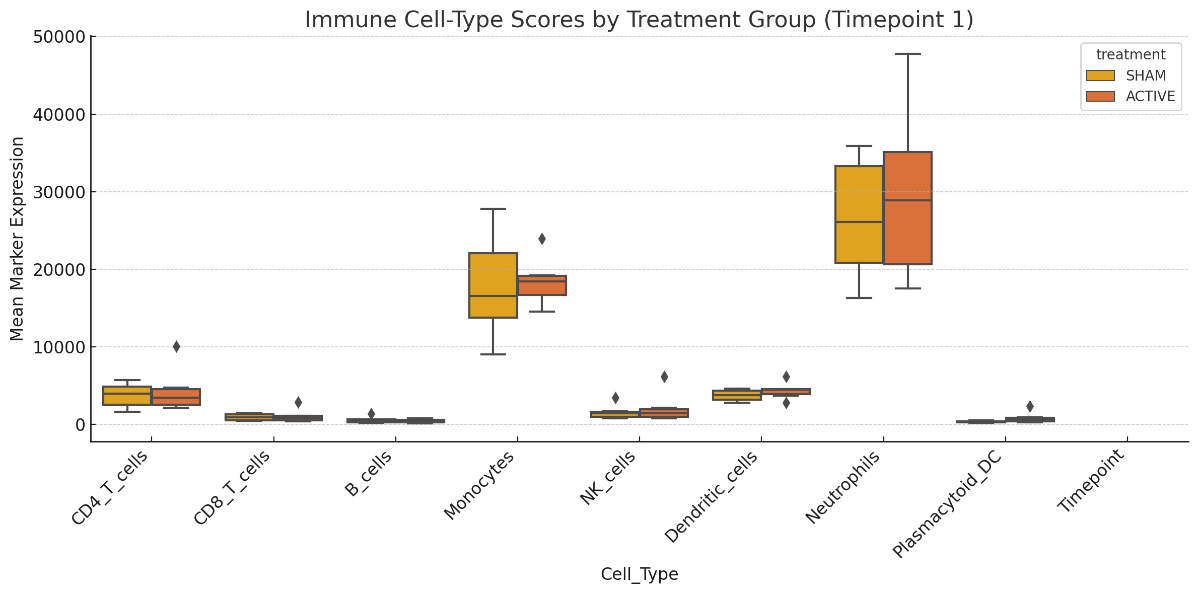


# Supplementary Figure 7. neutrophil types determined by CIBERSORT.

The CIBERSORT score consists of arbitrary units that reflect the absolute proportion of neutrophils in a mixture. Black bars represent median values.


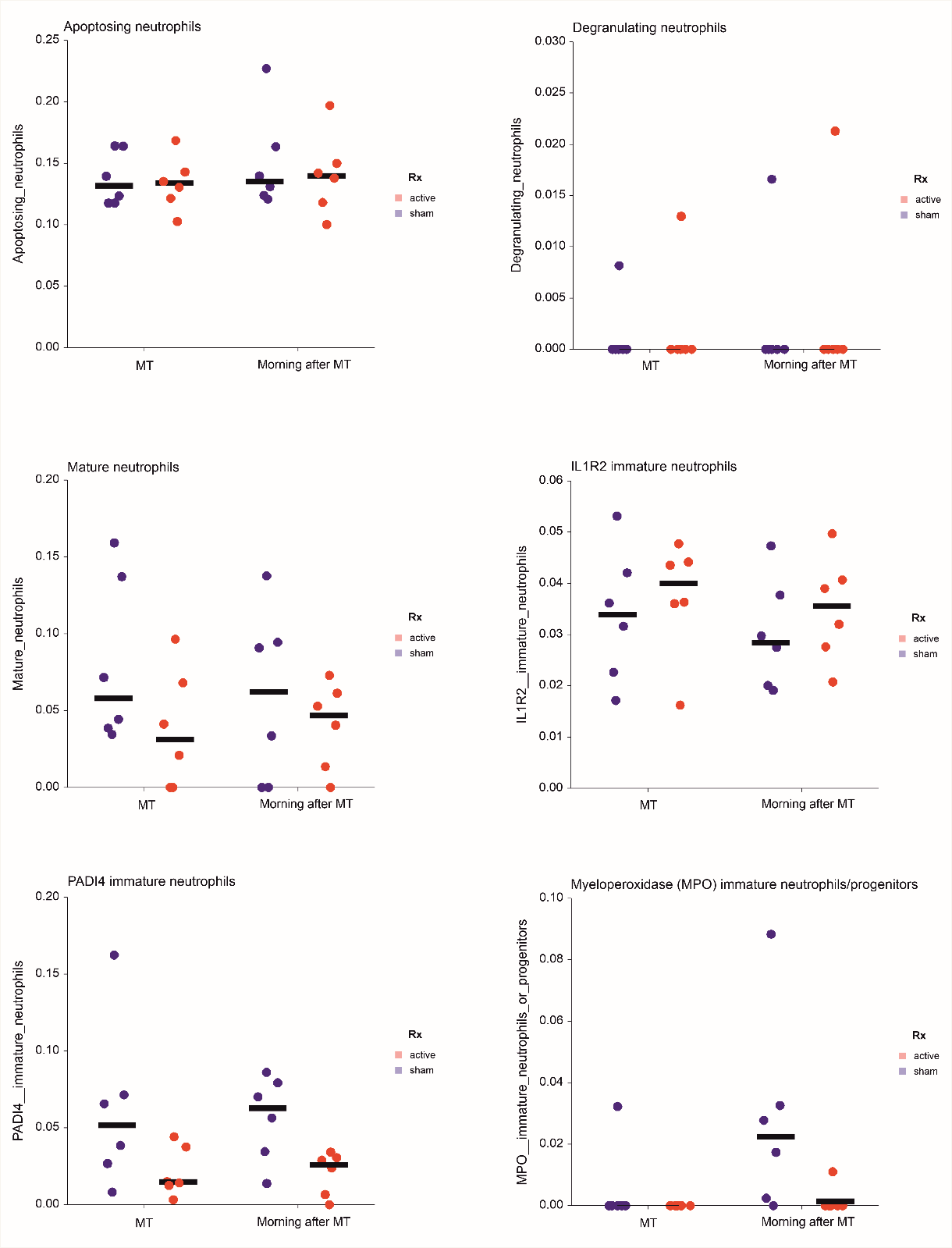


# Supplementary Figure 8. lymphocyte populations determined by CIBERSORT.

The CIBERSORT score consists of arbitrary units that reflect the absolute proportion of T and B lymphocytes in a mixture. Black bars represent median values.


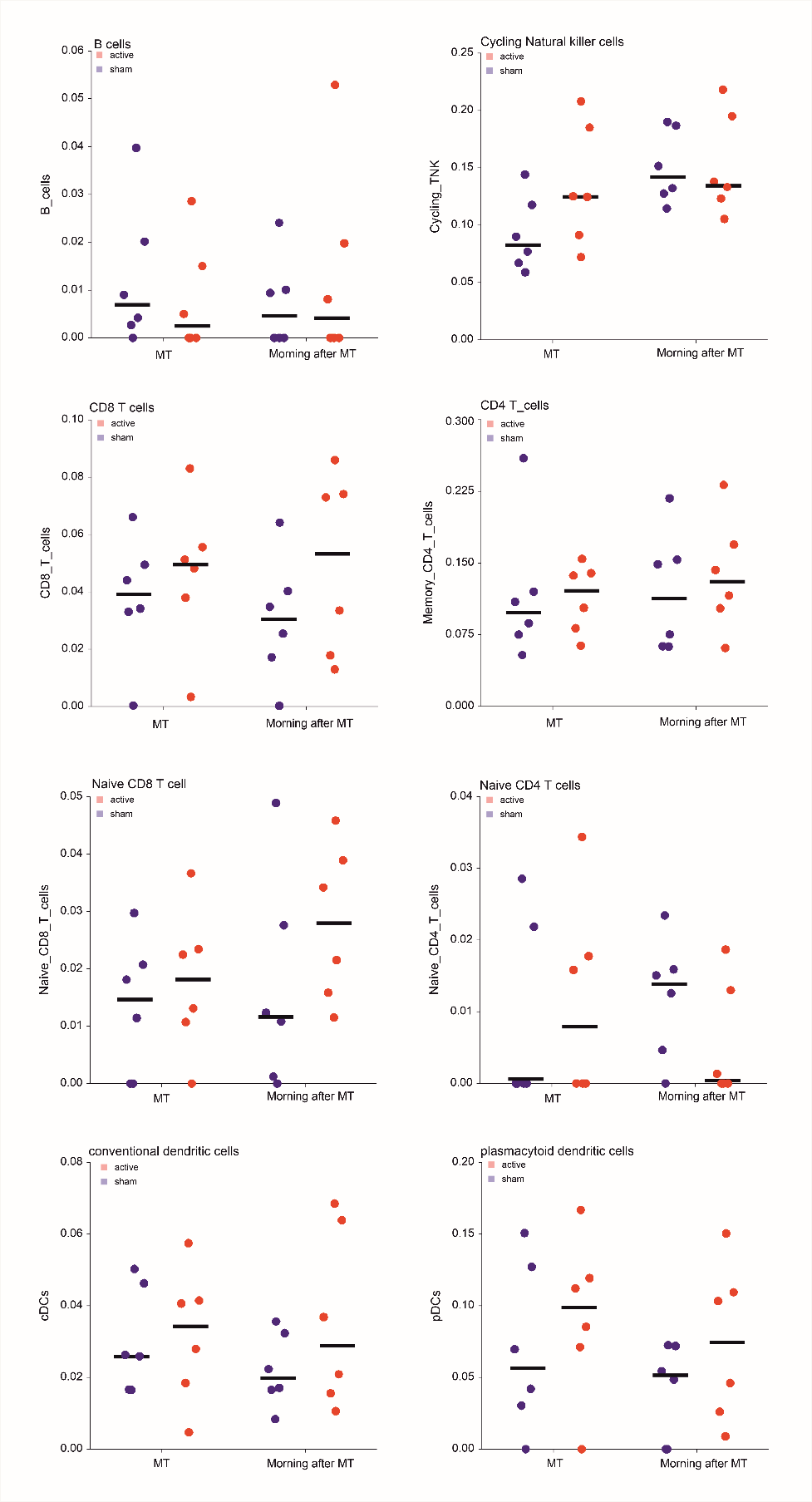


# Supplementary Figure 9. monocytes, eosinophils and platelets quantified by CIBERSORT.

The CIBERSORT score consists of arbitrary units that reflect the absolute proportion of monocytes, eosinophils and platelets in a mixture. Black bars represent median values.


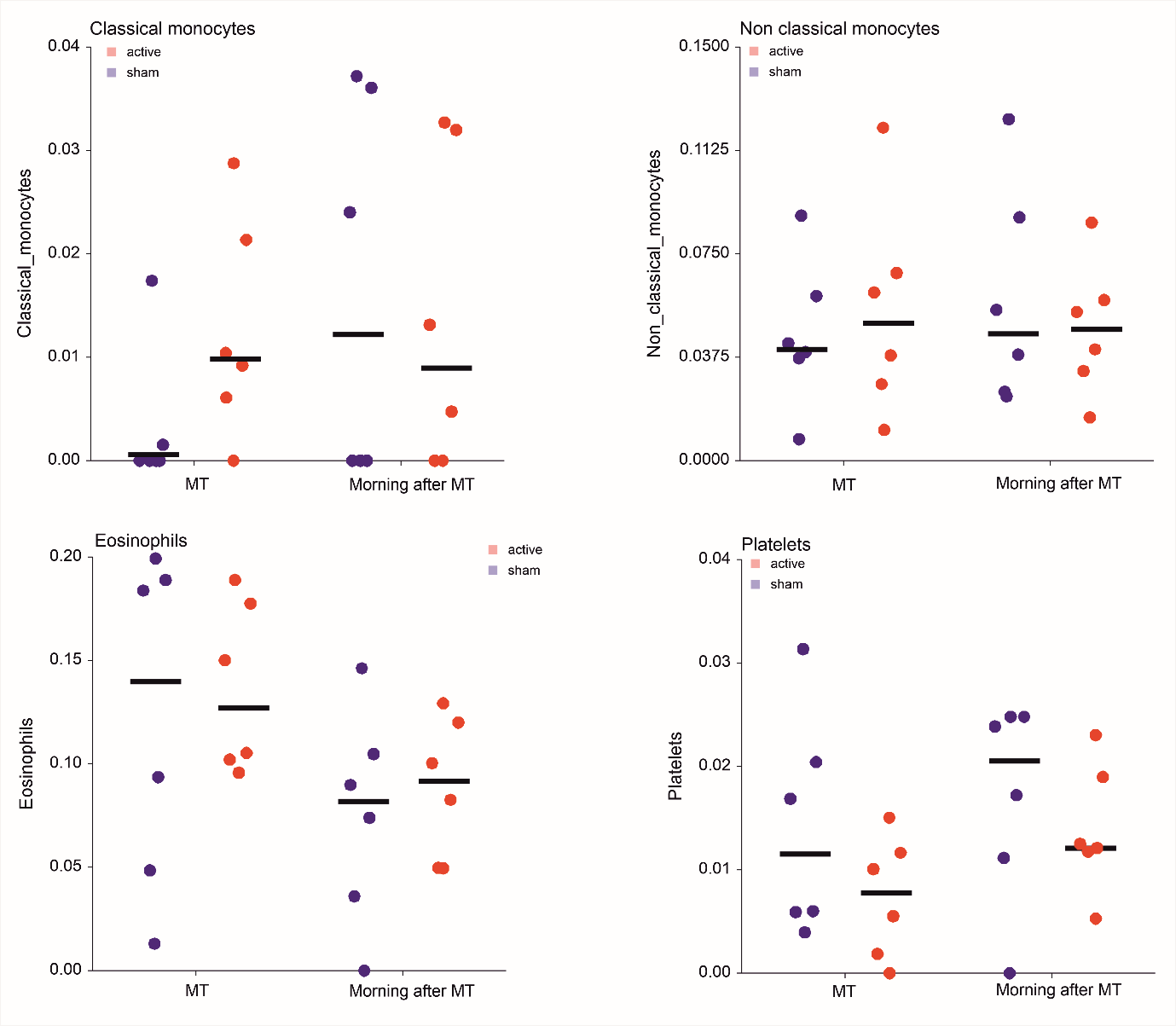


# Supplementary Figure 10. pathway analysis by KEGG

KEGG pathway map of differential gene expression, depicting genes with altered expression. Red boxes represent upregulated genes, while green boxes indicate downregulated genes. The analysis highlights activation of key inflammatory mediators and transcription factors involved in TNF-induced signaling cascades, including NF-κB, MAPK, and apoptosis-related pathways. Red highlighted genes = upregulation; GREEN = downregulation.

## TNF signaling


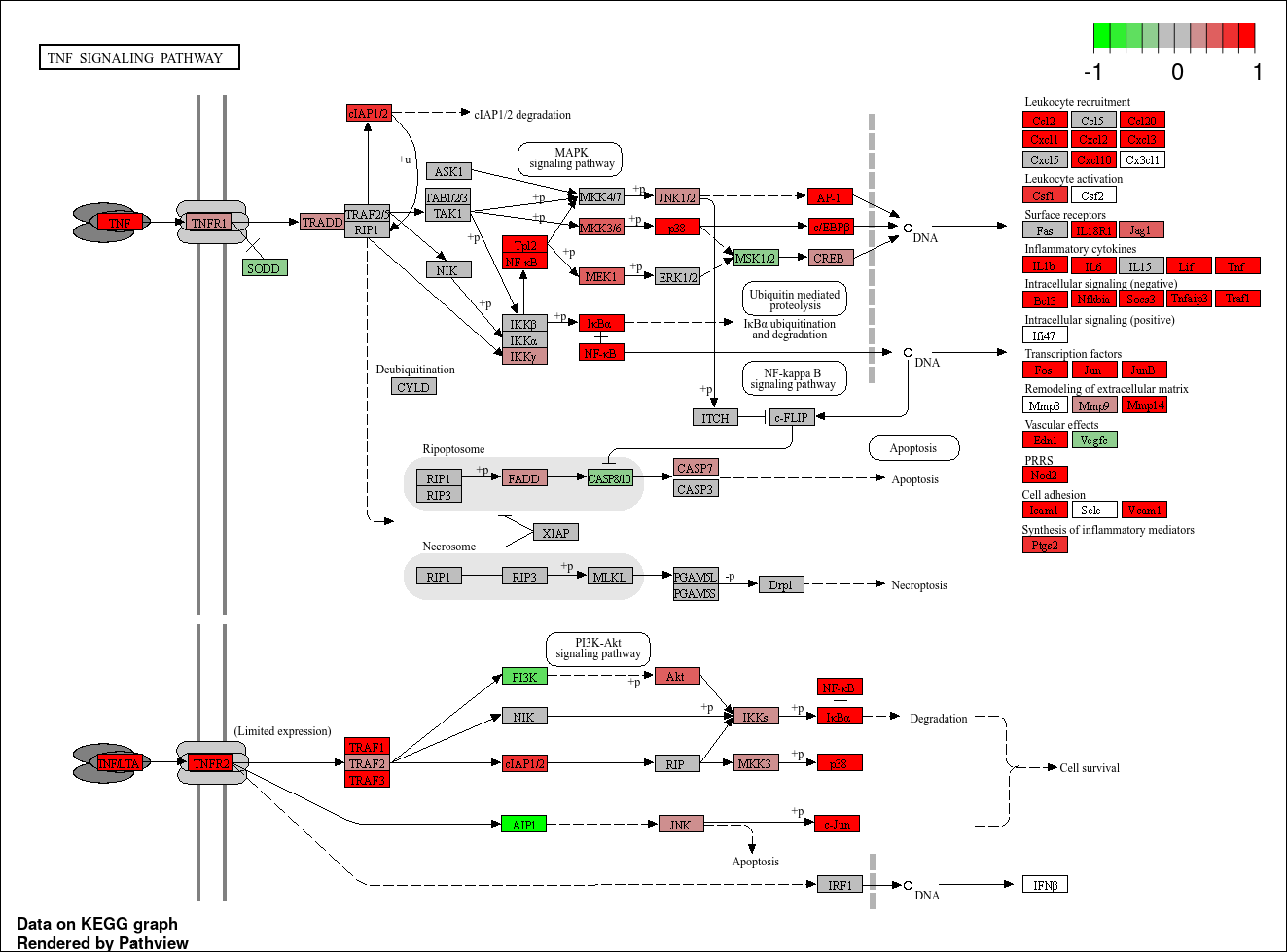


## IL-17 signalling pathway

**
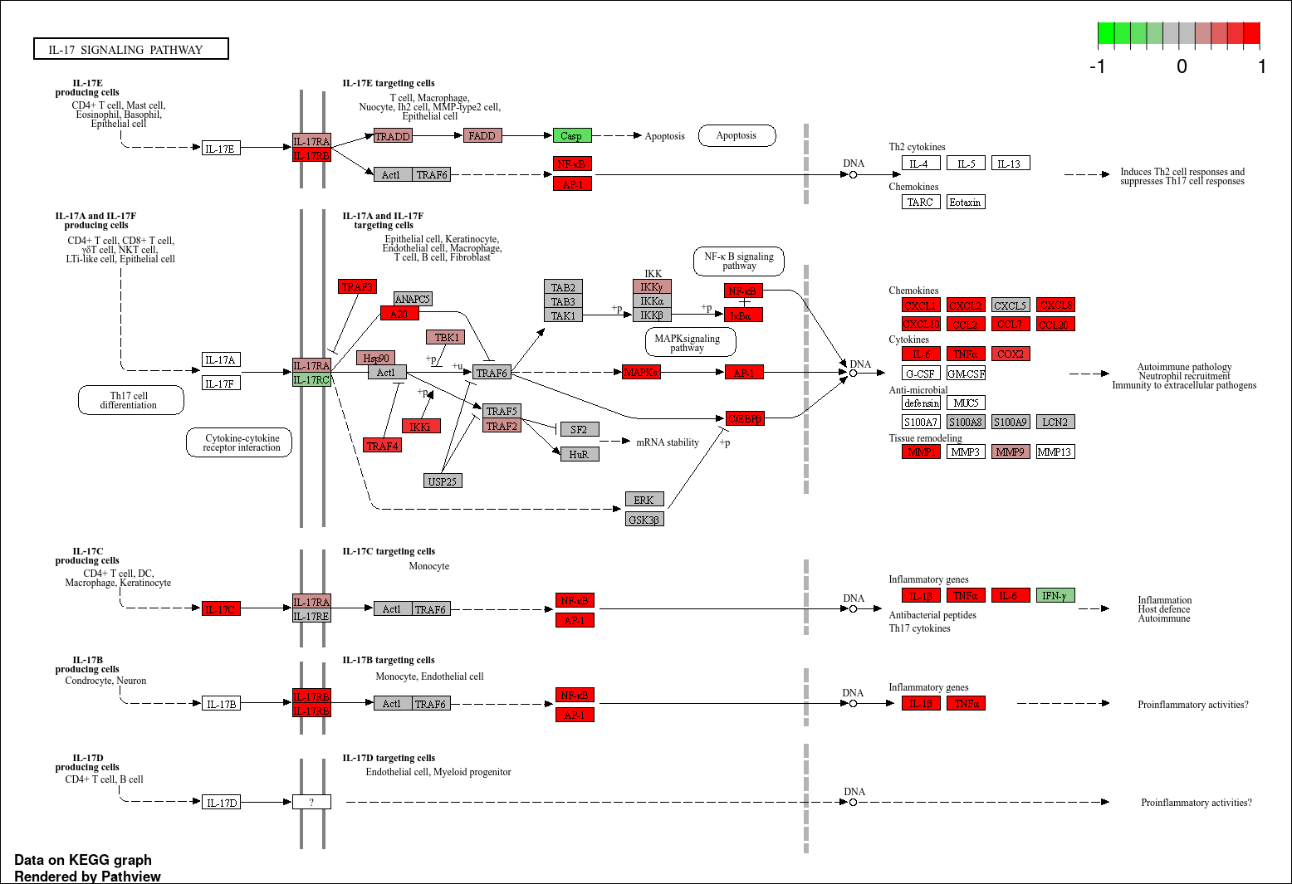
**

## **
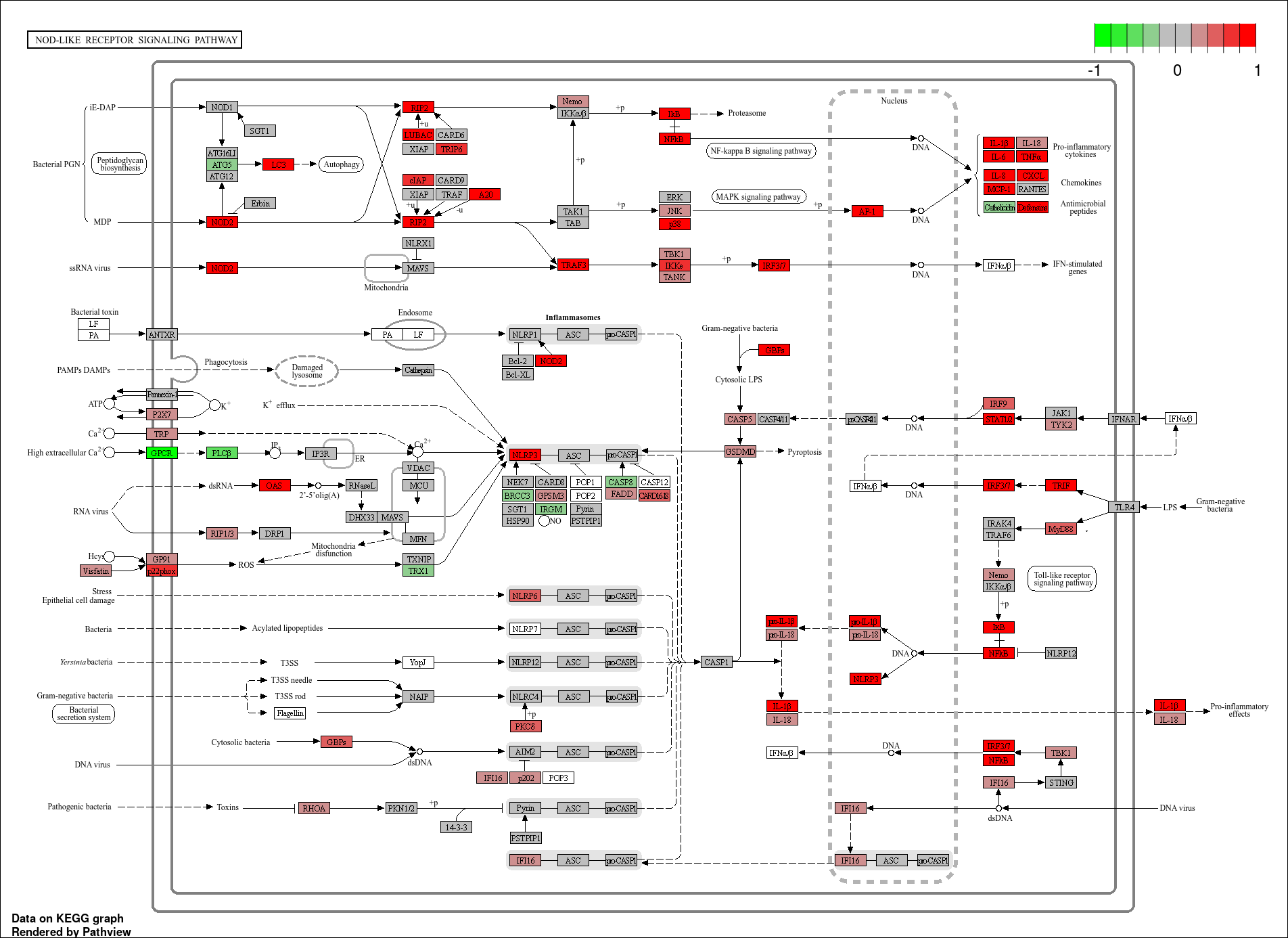
**NOD-like receptor signalling pathway

## **
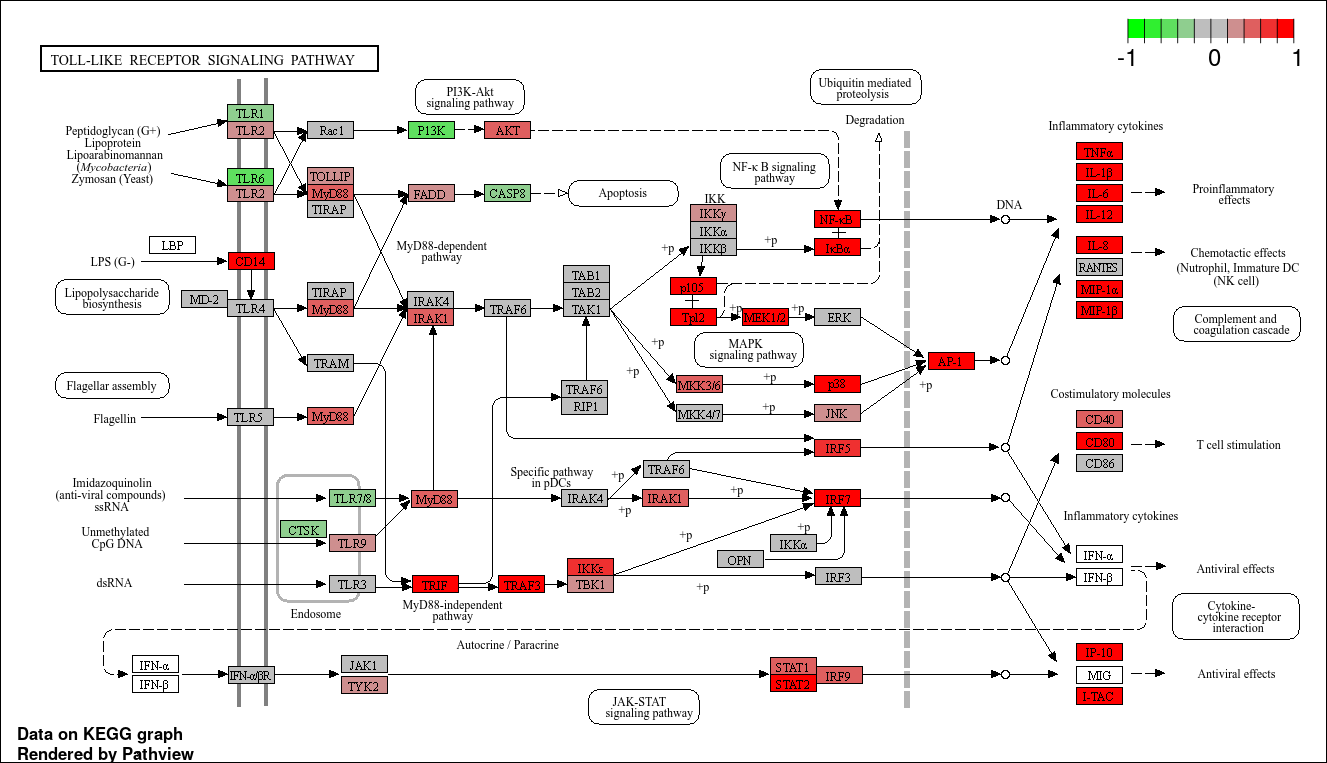
**Toll-like receptor signalling pathway

## Staphylococcus aureus infection

**
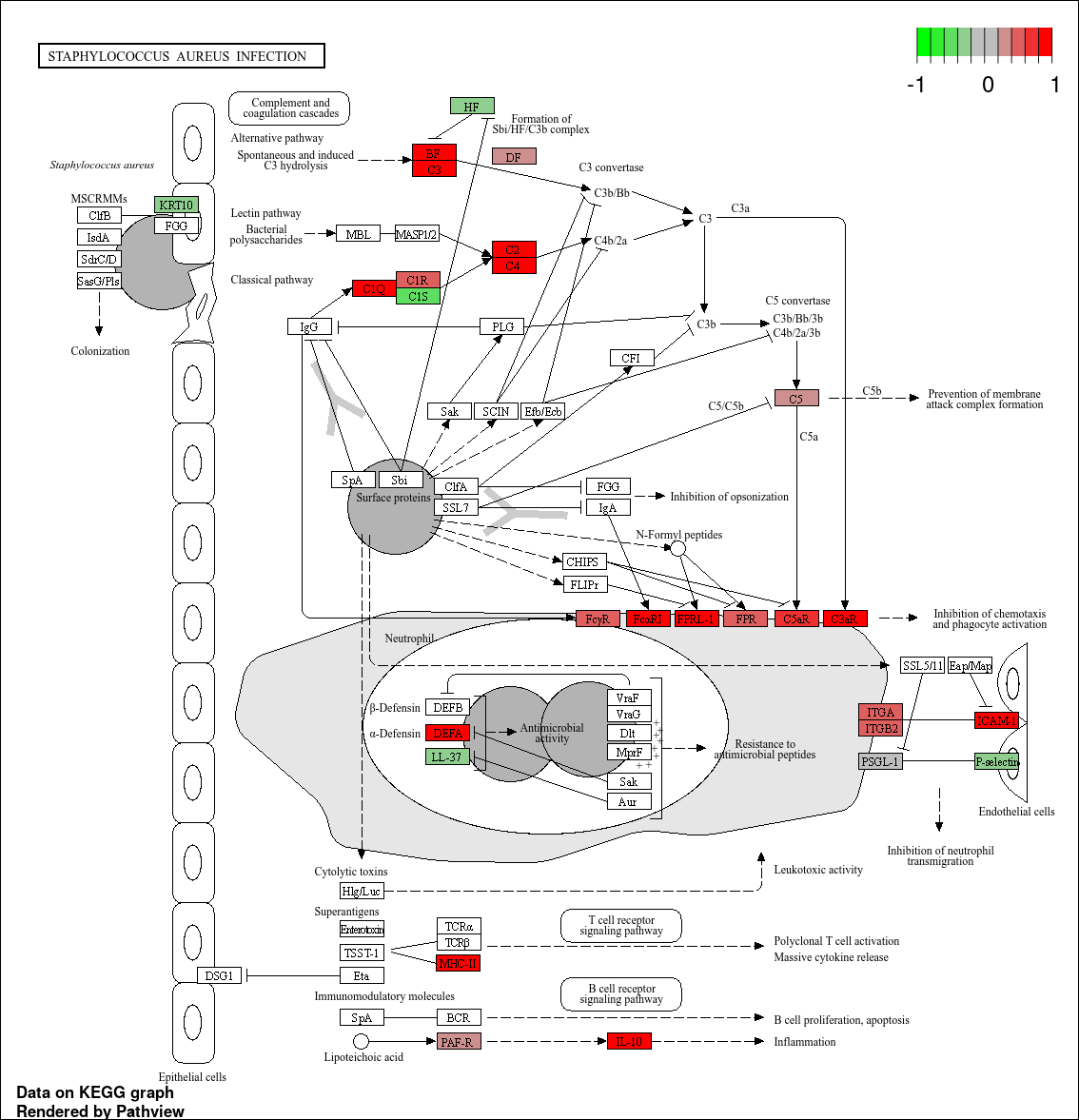
**

## Cytosolic DNA-sensing pathway

**
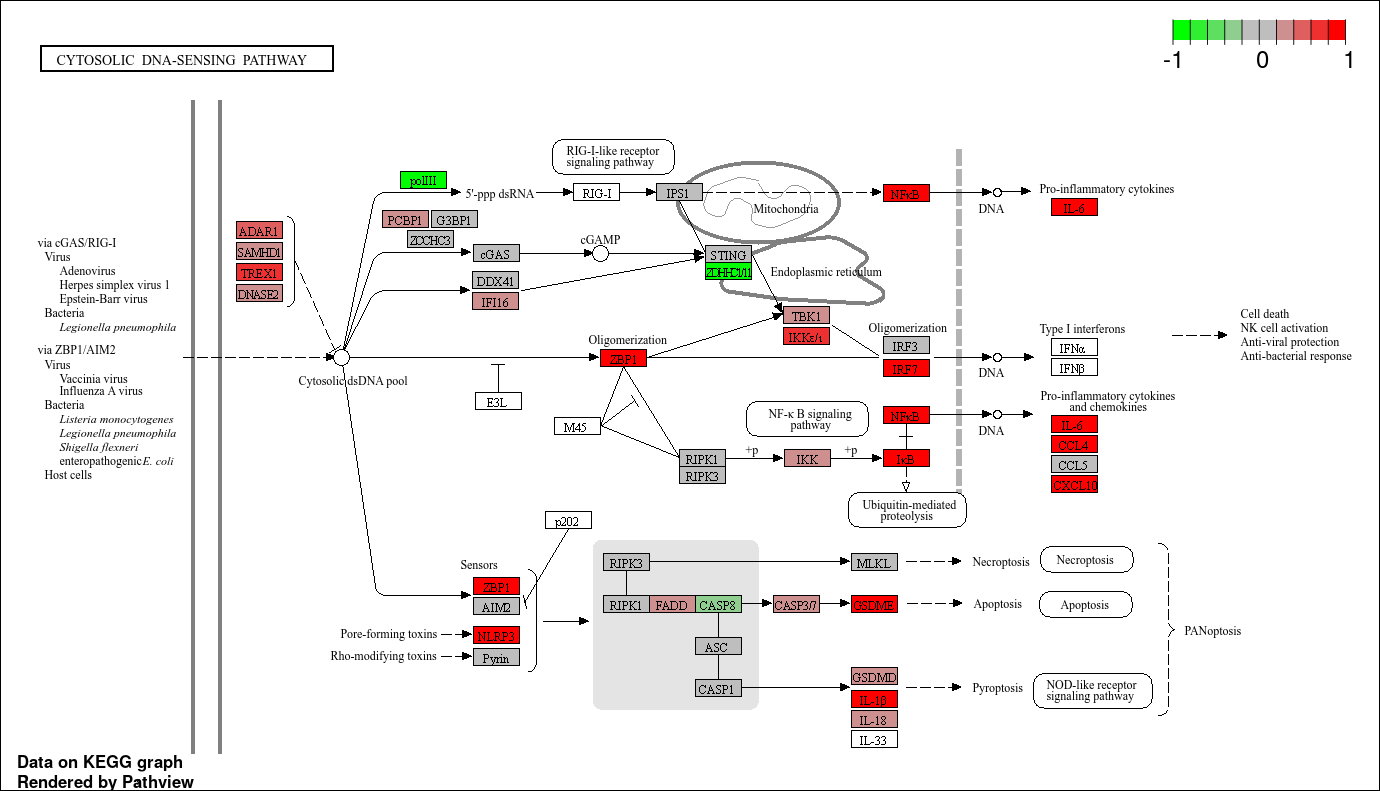
**

# Additional references

1. Hays SA, Rennaker RL, Kilgard MP. Targeting plasticity with vagus nerve stimulation to treat neurological disease. *Prog Brain Res*. 2013;207:275-299. doi:10.1016/B978-0-444-63327-9.00010-2

2. Kwong PWH, Ng GYF, Chung RCK, Ng SSM. Bilateral Transcutaneous Electrical Nerve Stimulation Improves Lower-Limb Motor Function in Subjects With Chronic Stroke: A Randomized Controlled Trial. *J Am Heart Assoc*. 2018;7(4):e007341. doi:10.1161/JAHA.117.007341

3. Farmer AD, Strzelczyk A, Finisguerra A, et al. International Consensus Based Review and Recommendations for Minimum Reporting Standards in Research on Transcutaneous Vagus Nerve Stimulation (Version 2020). *Front Hum Neurosci*. 2020;14:568051. doi:10.3389/fnhum.2020.568051

4. Patel ABU, Weber V, Gourine AV, Ackland GL. The potential for autonomic neuromodulation to reduce perioperative complications and pain: a systematic review and meta-analysis. *Br J Anaesth*. 2022;128(1):135-149. doi:10.1016/j.bja.2021.08.037

5. Mena L, Pintos S, Queipo NV, Aizpúrua JA, Maestre G, Sulbarán T. A reliable index for the prognostic significance of blood pressure variability. *J Hypertens*. 2005;23(3):505-511. doi:10.1097/01.hjh.0000160205.81652.5a

6. Heart rate variability: standards of measurement, physiological interpretation and clinical use. Task Force of the European Society of Cardiology and the North American Society of Pacing and Electrophysiology. *Circulation*. 1996;93(5):1043-1065.

7. Burr RL, Cowan MJ. Autoregressive spectral models of heart rate variability: Practical issues. *Journal of Electrocardiology*. 1992;25:224-233. doi:10.1016/0022-0736(92)90108-C

8. Taylor JA, Carr DL, Myers CW, Eckberg DL. Mechanisms underlying very-low-frequency RR-interval oscillations in humans. *Circulation*. 1998;98(6):547-555. doi:10.1161/01.cir.98.6.547

9. Guzzetti S, La Rovere MT, Pinna GD, et al. Different spectral components of 24 h heart rate variability are related to different modes of death in chronic heart failure. *Eur Heart J*. 2005;26(4):357-362. doi:10.1093/eurheartj/ehi067

10. Goldstein DS, Bentho O, Park MY, Sharabi Y. LF power of heart rate variability is not a measure of cardiac sympathetic tone but may be a measure of modulation of cardiac autonomic outflows by baroreflexes. *Exp Physiol*. 2011;96(12):1255-1261. doi:10.1113/expphysiol.2010.056259

11. Pagani M, Lombardi F, Guzzetti S, et al. Power spectral analysis of heart rate and arterial pressure variabilities as a marker of sympatho-vagal interaction in man and conscious dog. *Circ Res*. 1986;59(2):178-193. doi:10.1161/01.res.59.2.178

12. Newman AM, Steen CB, Liu CL, et al. Determining cell type abundance and expression from bulk tissues with digital cytometry. *Nat Biotechnol*. 2019;37(7):773-782. doi:10.1038/s41587-019-0114-2

13. Kwok AJ, Allcock A, Ferreira RC, et al. Neutrophils and emergency granulopoiesis drive immune suppression and an extreme response endotype during sepsis. *Nat Immunol*. 2023;24(5):767-779. doi:10.1038/s41590-023-01490-5
